# Identifying the structure of Zn-N_2_ active sites and structural activation

**DOI:** 10.1038/s41467-019-10622-1

**Published:** 2019-06-13

**Authors:** Feng Li, Yunfei Bu, Gao-Feng Han, Hyuk-Jun Noh, Seok-Jin Kim, Ishfaq Ahmad, Yalin Lu, Peng Zhang, Hu Young Jeong, Zhengping Fu, Qin Zhong, Jong-Beom Baek

**Affiliations:** 1 School of Energy and Chemical Engineering/Center for Dimension-Controllable Organic Frameworks, Ulsan National Institute of Science and Technology (UNIST), 50 UNIST, Ulsan, 44919 South Korea; 2 Jiangsu Key Laboratory of Atmospheric Environment Monitoring and Pollution Control, School of Environmental Science and Engineering, Nanjing University of Information Science and Technology, 219 Ningliu, 210044 Nanjing, Jiangsu P.R. China; 3 CAS Key Laboratory of Materials for Energy Conversion, Hefei National Laboratory for Physical Sciences at Microscale, National Synchrotron Radiation Laboratory, University of Science and Technology of China, 96 Jinzhai, 230026 Hefei, Anhui P.R. China; 4 Institute for Advanced Materials, School of Materials Science and Engineering, Jiangsu University, 301 Xuefu Road, 212013 Zhenjiang, P.R. China; 5 UNIST Central Research Facilities, Ulsan National Institute of Science and Technology (UNIST), 50 UNIST, Ulsan, 44919 South Korea; 6 School of Chemical Engineering, Nanjing University of Science and Technology, Xiaolingwei Street No. 200, 210094 Nanjing, P.R. China

**Keywords:** Catalytic mechanisms, Electrocatalysis, Electrocatalysis

## Abstract

Identification of active sites is one of the main obstacles to rational design of catalysts for diverse applications. Fundamental insight into the identification of the structure of active sites and structural contributions for catalytic performance are still lacking. Recently, X-ray absorption spectroscopy (XAS) and density functional theory (DFT) provide important tools to disclose the electronic, geometric and catalytic natures of active sites. Herein, we demonstrate the structural identification of Zn-N_2_ active sites with both experimental/theoretical X-ray absorption near edge structure (XANES) and extended X-ray absorption fine structure (EXAFS) spectra. Further DFT calculations reveal that the oxygen species activation on Zn-N_2_ active sites is significantly enhanced, which can accelerate the reduction of oxygen with high selectivity, according well with the experimental results. This work highlights the identification and investigation of Zn-N_2_ active sites, providing a regular principle to obtain deep insight into the nature of catalysts for various catalytic applications.

## Abstract (web summary)

Identification of active sites is one of the main obstacles to rational design of catalysts for scientific and industrial applications. Here, the authors demonstrate the synthesis and structural identification of Zn based active sites, as well as the related structural activation for oxygen species.

## Introduction

Active sites are at the heart of catalysts, while the nature of active sites plays a key role in the performance of catalysts^1–6^. During the past decade, the active sites of transition metal–nitrogen–carbon (TMNC) catalysts have not been well identified and are simply defined as TM-N_*x*_ based on information from X-ray photoelectron spectroscopy (XPS)^7–11^. Such rough recognition of active sites leads to ambiguous understanding of the reaction mechanisms occurring on the surface of the catalysts, as well as stagnation of the development of rational catalyst design strategies. Recently, synchrotron radiation-based extended X-ray absorption fine structure (EXAFS) spectrum analysis, along with experimental characterization and theoretical simulation, has gradually been introduced to identify the geometric structures of active sites^12–15^. Despite this progress, however, the more structurally sensitive X-ray absorption near edge structure (XANES) spectrum analysis has been neglected. To obtain fundamental understanding of active sites and catalytic mechanisms, identifying the electronic and geometric structures of active sites with both XANES and EXAFS spectra is still highly desired.

Among various TMNC catalysts, zinc (Zn)-based materials, due to the low sublimation temperature, have been difficult to achieve and little progress has been made. After the removal of metallic Zn by sublimation, Zn-containing materials are frequently used as precursors to produce porous nitrogenated carbon for various electrochemical applications or Zn-free single atom catalysts^12,15–19^. However, none of these can maintain the presence of elemental Zn, not to mention the relevant active sites. Recently, single Zn atoms have been stabilized on carbon black (ZnN_*x*_/C), with Zn-N_4_ as the structure of the active sites^20,21^. However, the active sites have been investigated simply by EXAFS, without XANES spectrum analysis, making identification of active site structures and ensuing reaction mechanisms ambiguous and unreliable. It is still a great challenge to construct and identify Zn-based active sites, as well as to discern structural activation for catalytic applications.

Herein we demonstrate the synthesis and structural identification of Zn-based active sites, as well as the related structural activation for oxygen species. Combined EXAFS and XANES spectra analysis confirmed Zn-N_2_ as the structure of the active sites. First-principles density-functional theory (DFT) calculations reveal that the O-O bond stretching of adsorbed O_2_ (*O_2_) and OOH (*OOH) on Zn-N_2_ active sites are significantly enhanced. The high degree of O-O bond stretching can accelerate the highly selective four-electron reduction of adsorbed oxygen on the surface of Zn-N_2_ active sites, which agrees well with the experimental results.

## Results

### Synthesis and structural characterization

**Fig. 1:**
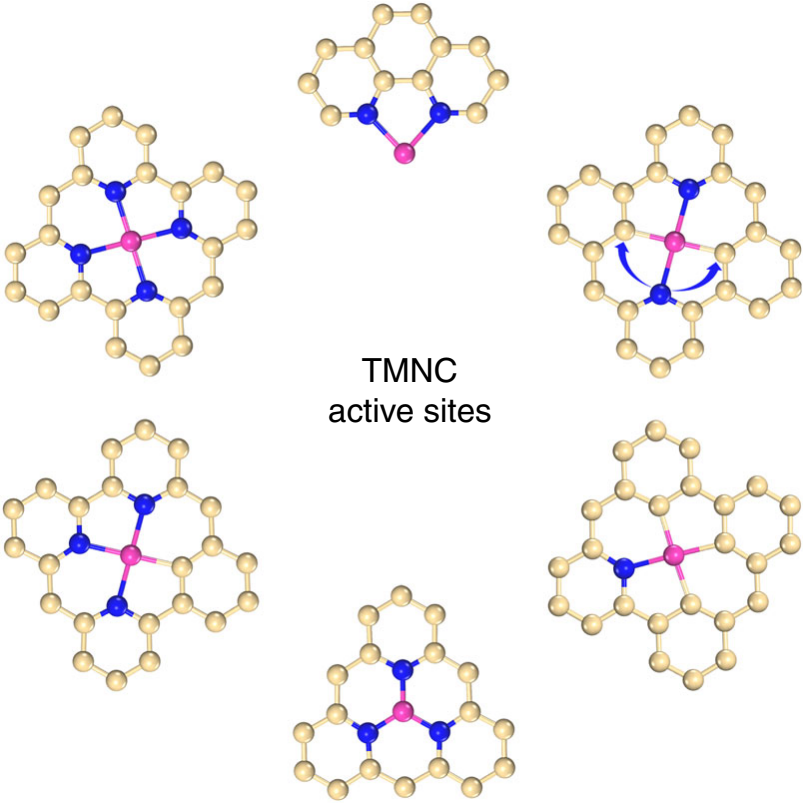
Schematic illustration of active site structures in transition metal–nitrogen–carbon system. Light yellow, blue and pink ivory balls represent carbon, nitrogen and transition metal atoms, respectively

Figure 1 shows typical structures of active sites of TMNC catalysts. With different coordination environments, the electronic and geometric structures of the active sites are largely different. Importantly, differences of the electronic and geometric structures can lead to diverse adsorption behaviours, which play key roles in the performance of catalysts. To begin with, Zn-based TMNC material (ZnNC) was prepared by thermal treatment of Zn-containing hybrid precursor in an argon atmosphere at 800 °C for 6 h (Supplementary Fig. 1). According to reported works, prolonged higher temperature pyrolysis can result in the vanish of Zn^22^.

The crystalline structure of ZnNC was investigated using high-power X-ray diffraction (HP-XRD) equipment (Supplementary Fig. 2). The two diffraction peaks of ZnNC at around 26° and 43° can be assigned to the (002) and (100) planes of graphitic carbon. No peaks of metallic Zn and Zn oxide were detected. The structural and chemical compositions of ZnNC were further studied by scanning electron microscopy (SEM), transmission electron microscopy (TEM) and Brunauer–Emmett–Teller (BET) specific surface area analysis (Supplementary Figs. 3–5). The polyhedral building blocks in ZnNC exhibited an average size of 50 nm and consisted of C, N and Zn elements. Importantly, the element mapping images showed that the C, N and Zn elements are uniformly distributed in ZnNC. The high-resolution scanning transmission electron microscope (STEM) images further confirm the graphitic morphology of ZnNC, with an absence of metal or metal oxide nanoparticles for Zn element.

The XPS technique was further introduced to investigate the detailed chemical compositions of Zn and N species (Supplementary Fig. 6). The two peaks at binding energies of 1021.7 and 1044.8 eV in the high-resolution Zn 2*p* XPS spectrum belong to Zn 2*p*_3/2_ and Zn 2*p*_1/2_ of the Zn^2+^ species. The high-resolution N 1*s* XPS spectrum confirmed the presence of four different N species, including pyridinic N (398.5 eV), pyrrolic N (400.1 eV), graphitic N (401.0 eV) and oxidized N (404.6 eV)^23^. The atomic concentrations of the various N species are 2.28 at%, 0.26 at%, 2.34 at% and 0.39 at%, respectively. In relation to pyrrolic N, the content of pyridinic N is significantly higher, which can be attributed to the higher thermal stability of pyridinic N. Generally, uniformly distributed pyridinic N plays an important role in the active sites of TMNC materials, which can stabilize TM atoms in the NC matrix by formation of TM-N bonds.

### Active site structure identification

**Fig. 2:**
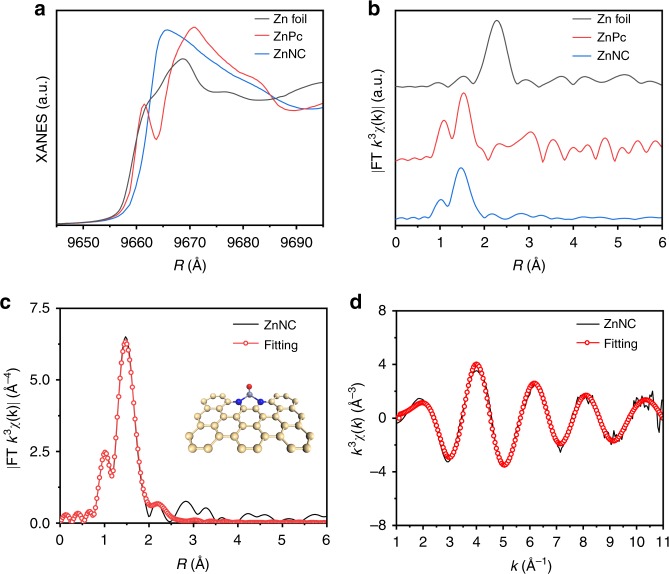
Structural analysis of ZnNC by X-ray absorption fine structure (XAFS) spectroscopy. **a** Zn *K*-edge X-ray absorption near edge structure spectra of Zn foil, ZnPc and ZnNC. **b** Fourier transform (FT) of the Zn *K*-edge extended XAFS (EXAFS) spectra of Zn foil, ZnPc and ZnNC. **c**, **d** Corresponding EXAFS fitting curve of ZnNC in *R* and *k* spaces, respectively. Insets are the schematic model of Zn-N_2_. Light yellow, blue, light purple and red ivory balls represent carbon, nitrogen, zinc and oxygen atoms, respectively

Synchrotron radiation-based X-ray absorption spectroscopy, with high sensitivity to electronic and geometric structures, was adopted for identification of the structure of active sites. Figure 2a shows the *K*-edge XANES spectrum of ZnNC, with ZnPc and Zn foil as the references. The location of the absorption edge for ZnNC reveals that the oxidation state of the Zn atom is between 0 and + 2. The Fourier-transformed (FT) k^3^-weighted EXAFS spectrum of ZnNC exhibits a main peak at around 1.47 Å, corresponding to Zn-N coordination. The possibility of Zn-C coordination was excluded by the following XANES spectrum simulation (Supplementary Fig. 7). In contrast to the case of Zn foil, no peak belonging to Zn-Zn bond was detected at around 2.2 Å (Fig. 2b). Based on the position of the absorption edge, the Zn-N_2_ model structure was proposed as the geometric structure of the ZnNC active sites and was further used to fit the corresponding Fourier transform (FT) *k*^3^-weighted EXAFS spectrum in both *R* and *k* spaces (Supplementary Table 1). As shown in Fig. 2c, d, the FT *k*^3^-weighted EXAFS spectrum of ZnNC can be well fitted, suggesting Zn-N_2_ configuration as the active sites of ZnNC.

**Fig. 3:**
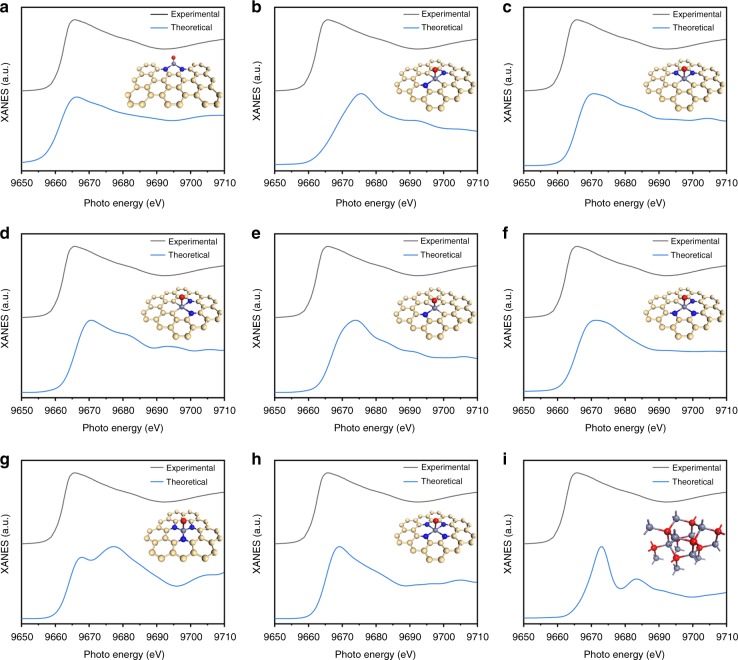
Theoretical calculation of X-ray absorption near edge structure (XANES) spectra. **a**–**i** Comparison of Zn *K*-edge XANES spectra of ZnNC and theoretical XANES spectra calculated with different active site structures. Insets are corresponding schematic models of the active site structures. Light yellow, blue, light purple and red ivory balls represent carbon, nitrogen, zinc and oxygen atoms, respectively

Compared with the EXAFS spectrum, the XANES spectrum is more sensitive and is thus essential for the identification of the structure of active sites. To confirm the structure of the active sites, XANES spectra were further simulated with typical structures of TMNC active sites (Fig. 3). Figure 3a shows the Zn *K*-edge XANES spectrum of ZnNC and the theoretical XANES spectrum based on the structure of the proposed Zn-N_2_ model. The calculated spectrum can reproduce identical features and matches well with the experimental results. Compared with the experimental spectrum, the calculated spectra for the structures of Zn-N_1_C_3_, Zn-N_2_C_2_, Zn-N_3_C_1_, Zn-N_4_ models and ZnO, which exhibit more positive absorption edges, are strikingly different. Although the theoretical spectrum for the structure of the Zn-N_3_ model shows an adsorption edge similar to that of the experimental spectrum, a corresponding strong feature at around 9677.3 eV interrupts this agreement. In the energy range of 9669–9677 eV, no obvious other models related intensity enhancing was observed in the experimental spectrum, revealing the high homogeneity of Zn-N_2_ model.

**Fig. 4:**
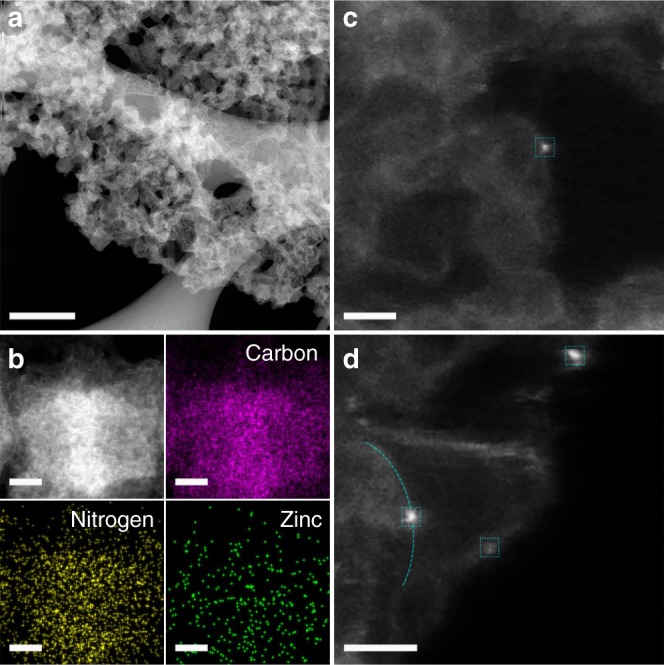
Structural characterization of ZnNC by atomic resolution scanning transmission electron microscopy (STEM). **a**, **b** Low-resolution STEM and element mapping images of ZnNC. **c**, **d** Atomic resolution STEM image of ZnNC. The Zn single atoms indexed with cyan blue square, confirming that the Zn single atoms are located on the edge of the nitrogenated carbon matrixes. Scale bar: **a** 200 nm; **b** 10 nm; **c**, **d** 1 nm

This result is consistent well with the optimized typical structures of Zn active sites (Supplementary Fig. 8). As shown, the configurations of Zn-N_1_C_3_, Zn-N_2_C_2_, Zn-N_3_C_1_, Zn-N_3_ and Zn-N_4_ are instable, in which the Zn atoms have struggled out of the nitrogenated carbon matrix planes. The exposed Zn atoms can be easily reduced into metallic Zn by the surrounded adjacent carbon species. Unlike other transition metals, such as Fe and Co, the formed metallic Zn can be further removed steadily via sublimation at high temperature. In this regard, Zn-N_2_ configuration on the edge sites of the nitrogenated carbon matrix planes exhibits the most stable configuration, which faces the minimum adjacent carbon species and has the most opportunity to survive at high temperature. The following atomic resolution STEM images in Fig. 4c, d further confirmed the sole edge site location of Zn single atoms at the nitrogenated carbon matrixes. No Zn-N_1_C_3_, Zn-N_2_C_2_, Zn-N_3_C_1_, Zn-N_3_ and Zn-N_4_ configuration-related location site for Zn single atoms was observed. At the meantime, the nitrogenated carbon matrixes are very small in size, which can provide abundant edge sites for the maximum coordinating of Zn single atoms.

**Fig. 5:**
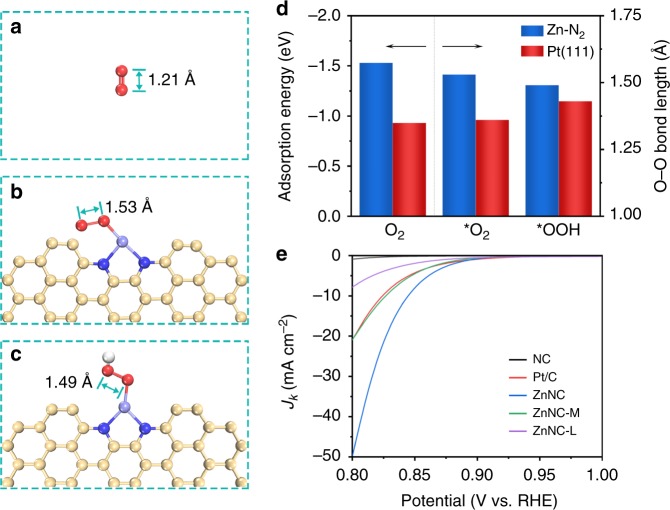
Theoretical and experimental investigations of oxygen species activation. **a** Molecular structure of gaseous O_2_. **b**, **c** O_2_ and OOH adsorption configurations on Zn-N_2_ active site. **d** O_2_ adsorption energy, O-O bond lengths of adsorbed O_2_ and OOH on Zn-N_2_ active site and Pt(111) surface. **e** Polarization curves of ZnNC with different Zn concentrations and Pt/C. Light yellow, blue, light purple, red and white ivory balls represent carbon, nitrogen, zinc, oxygen and hydrogen atoms, respectively

Overall, combining EXAFS with XANES spectra, the structure of the active sites for ZnNC can be confirmed without ambiguity to be the Zn-N_2_ structure. Structural identification of the active sites is highly beneficial for the analysis of structural activation to determine catalytic behaviours on the surfaces of catalysts.

### Theoretical study of the structural activation

Electrochemical reduction of O_2_ to H_2_O in aqueous medium via a robust four-electron pathway has been pursued for decades by TMNC catalysts. Active site structural activation for O-O bond stretching in *O_2_ and *OOH, and O_2_ adsorption plays an important role in the oxygen reduction process^23–25^.

To obtain fundamental insight into structural activation for oxygen species on Zn-N_2_ active sites, first-principles DFT calculations were conducted. The Pt(111) surface was also investigated as a reference (Supplementary Fig. 9). Figure 5a shows the optimized gaseous O_2_ molecule, which exhibits an O-O bond length of 1.21 Å. Figure 5b, c show the optimized configurations of O_2_ and OOH adsorbed on the Zn-N_2_ active site. Importantly, the bond lengths for *O_2_ and *OOH are 1.53 and 1.49 Å, respectively, which are 1.26 and 1.23 times that of the gaseous O_2_ molecule. The degree of O-O bond stretching of *O_2_ and *OOH on Zn-N_2_ active sites is also higher than that on Pt(111). With a higher degree of O-O bond stretching, much easier breaking of O-O bonds can be expected. The higher degree of O-O bond stretching can accelerate the rate of selective four-electron oxygen reduction and suppress the formation H_2_O_2_ via an inefficient two-electron pathway.

On the other hand, the O_2_ adsorption energies on Zn-N_2_ active sites and Pt(111) are –1.53 and –0.93 eV, respectively (Supplementary Table 2). The high O_2_ adsorption energy on the Zn-N_2_ active sites is more favoured by oxygen reduction, which can allow fast reactant supply for the subsequent reaction steps. Although the carbon atoms adjacent pyridinic N are also constantly considered as the active sites, the O_2_ adsorption energies on the carbon atoms are much lower, leading to a quite sluggish oxygen reduction pathway^23^. Meanwhile, the oxygen reduction is more efficient at an overpotential of 0.4 V (Supplementary Fig. 10).

According to the identified structure of active sites and the structural activation of oxygen species, we believe that Zn-N_2_ active sites incorporating ZnNC exhibit favourable catalytic behaviour for oxygen reduction.

### Experimental evaluation on ORR catalytic activity

The catalytic behaviours of ZnNCs for oxygen reduction were further evaluated in an oxygen-saturated 0.1 M aq. KOH solution. The contents of Zn in ZnNC, ZnNC-M and ZnNC-L were around 4.03 wt%, 3.68 wt% and 2.81 wt%, respectively (Supplementary Figs 11 and 12). NC and Pt/C were compared under the same conditions (Supplementary Fig. 13). Figure 5e shows the potential dependence of the kinetic current density (*J*_k_) on ZnNC with different Zn concentrations and Pt/C. *J*_k_ was calculated using the Koutecky–Levich equation. Without Zn active sites, NC exhibits poor catalytic activity in the potential range of 0.8–1.0 V vs. the reversible hydrogen electrode (RHE). *J*_k_ values at 0.85 V for ZnNC-L, ZnNC-M and ZnNC are –1.9, –4.5 and –7.9 mA cm^–2^, respectively, while that for Pt/C is around –4.3 mA cm^–2^. Compared with Pt/C, ZnNC exhibits much higher mass activity (Supplementary Fig. 14). Following the increasing Zn concentration, ZnNC demonstrates significantly enhanced catalytic activity towards oxygen reduction. Meanwhile, the decreasing Tafel slopes (81, 55 and 27 mV dec^–1^) confirm that the corresponding reaction kinetics also become much faster (Supplementary Fig. 15). With a smaller Tafel slope and a more positive half-wave potential (*E*_1/2_; 0.857 V), ZnNC exhibits catalytic activity superior to that of other decent catalysts for oxygen reduction reaction^8,26–32^ (Supplementary Table 3).

The selectivity of ZnNC was further detected by monitoring the H_2_O_2_ yield through rotating ring–disk electrode (RRDE) measurements (Supplementary Fig. 16). By applying a constant potential to the Pt ring, the H_2_O_2_ yield for ZnNC was revealed to be 1.35–2.93% in the potential range of 0.5–0.7 V, which was merely half of that for Pt/C (3.16–4.61%). According to the ring and disk current, the electron transfer number (*n*) of ZnNC was further calculated and found to be 3.97, confirming an efficient four-electron oxygen reduction pathway. The good stability of ZnNC was also demonstrated in the results of a methanol poisoning test and long-term cycling tests in oxygen-saturated 0.1 M aq. KOH solution (Supplementary Fig. 17).

The demonstrated high catalytic activity and selectivity of Zn-N_2_ active sites for electrochemical reduction of O_2_ agree well with the theoretical calculations based on the structural activation of *O_2_ and *OOH. On the other hand, owing to the stronger OH adsorption of Zn-N_2_ active sites, the theoretical onset potential would be smaller than that of Pt(111). The result is different from the relative performance of ZnNC and Pt/C and requires study in the future.

## Discussion

In summary, Zn-N_2_ active sites have been achieved and identified by both EXAFS and XANES spectra. Theoretical calculations reveal that the structural activation of oxygen species on Zn-N_2_ active sites is favoured by the selective oxygen reduction, which is confirmed by the experimental results. This work not only achieves the preparation and identification of Zn-N_2_ active sites but also provides a regular principle to obtain deep insight into the nature of catalysts for various catalytic applications.

## Methods

### Synthesis of ZnNC

As a typical synthesis, Zn(CH_3_COO)_2_ (2 mmol), 2-methylimidazole (8 mmol) and CNT (5 mg) were dissolved in methanol (80 ml). Each mixture was sonicated for 20 min. The above solutions were mixed quickly and aged at ambient conditions for 24 h. The resultant precipitates were collected and dried in an oven under reduced pressure. The collected precursor was annealed at 800 °C for 6 h in an argon atmosphere with a fast argon flow rate of 1.5 L min^–1^, denoted as ZnNC. Sample synthesized with 15-h thermal treatment was denoted as ZnNC-M. ZnNC etched with 5% HCl for 4 h was denoted as ZnNC-L. For the NC synthesis, trimesic acid (0.1 g) was mixed with dicyandiamide (1.0 g) and annealed under the same conditions as ZnNC.

### Characterizations

A field-emission SEM (Nanonova 230, FEI, USA) was used to obtain the SEM images of the samples. A high-resolution TEM (JEM-2100F, JEOL, Japan) was used to get the TEM images of the samples. A high-power X-ray diffractometer (D/MAZX 2500 V/PC, Rigaku, Japan) was applied to obtain the XRD patterns. An X-ray photoelectron spectrometer (K-alpha, Thermo Fisher Scientific, UK) was introduced to conduct the XPS analysis. Nitrogen adsorption–desorption isotherms were used to analyse the specific surface area, using the BET method (BELSORP-max, BEL, Japan). XAFS test was conducted at Pohang Light Source (PLS-II) in Korea.

The catalytic performance was investigated on an electrochemical workstation (Ivium, Netherlands) with a typical three-electrode cell. The counter-electrode and reference electrode were graphite rod and Ag/AgCl (saturated KCl) electrodes, respectively. All the potentials were referenced vs. RHE. The commercial Pt/C was obtained from Alfa Aesar (platinum, nominally 20% on carbon Black HiSPEC 3000). Catalyst (4 mg) and Nafion solution (30 μL, Aldrich Chemical Inc.) were dispersed in 0.5 ml ethanol/isopropyl alcohol solution (1/3, v/v). Catalyst ink was obtained by ultrasonicating the solution for 30 min. Catalyst film for the electrochemical test was formed by dropping the ink (8.32 µL) onto RRDEs (4 mm in diameter). The loading amount of Pt/C and catalysts are 0.2 and 0.5 mg cm^–2^, respectively (Pt/C: 40 μg_Pt_ cm^−2^; ZnNC: 20 μg_Zn_ cm^−2^). Linear sweep voltammetry was conducted in 0.1 M aq. KOH solution (oxygen saturated). The scan rate and rotation speed were 5 mV s^−1^ and 1600 r.p.m., respectively. A constant potential of 0.3 V (vs. Ag/AgCl) was applied on the Pt ring. The percentage of HO_2_ and electron transfer number (*n*) were calculated by the disc, ring current and Pt ring collection efficiency. The kinetics current was calculated using Koutecky–Levich equation.

### Theoretical calculation

The Vienna Ab Initio Simulation Package (VASP) was used to conduct the DFT calculations, employing the Perdew–Burke–Ernzerhof-type gradient-corrected exchange-correlation potential. The cutoff energy for the plane-wave basis set was 630 eV. The ionic potentials were described by projector-augmented wave potentials. The atomic configurations were relaxed with residual forces <0.01 eV Å^−1^. The Zn *K*-edge XANES spectra calculations were carried on the optimized atomic structures of ZnNC active sites with the full-potential augmented plane wave+local orbitals method, considering core–hole correction.

## Supplementary information

Supplementary Information

## Data Availability

The data that support the findings of this study are available from the corresponding author upon reasonable request.
