# Supplementary material for: Identifying the structure of Zn-N2 active sites and structural activation
Source: Nat Commun. 2019 Jun 13;10:2623. doi: 10.1038/s41467-019-10622-1 (PMC6565687; doi:10.1038/s41467-019-10622-1)
Supplement: Supplementary file 1 — Supplementary Information [file 41467_2019_10622_MOESM1_ESM.pdf]

Supplementary Information

Identifying the structure of Zn-N_2 active sites and structural activation

By Li *et al*

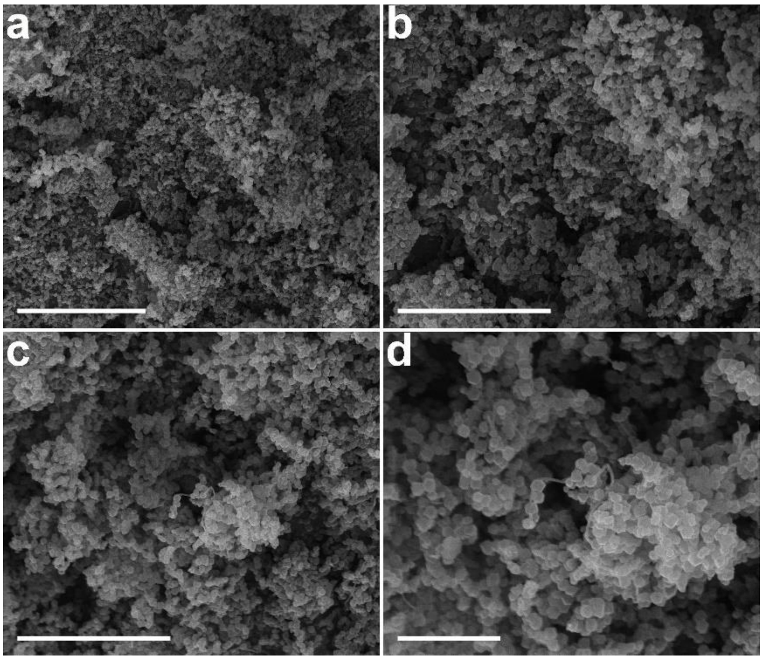

Supplementary Figure 1 | a-d, SEM images for precursor of ZnNC at different magnification. Scale bar: **a**, 5 μm; **b**, **c**, 3 μm; **d**, 1 μm.

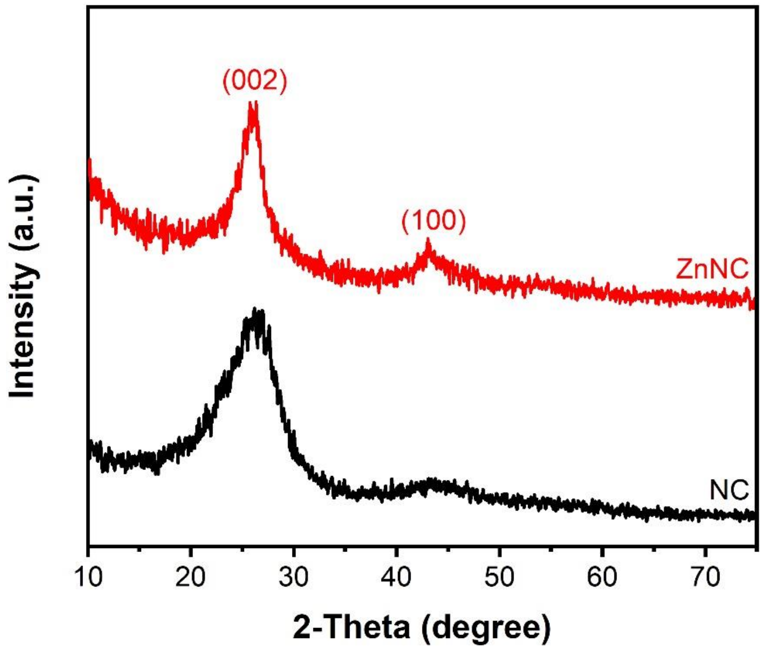

Supplementary Figure 2 | XRD patterns for ZnNC and NC.

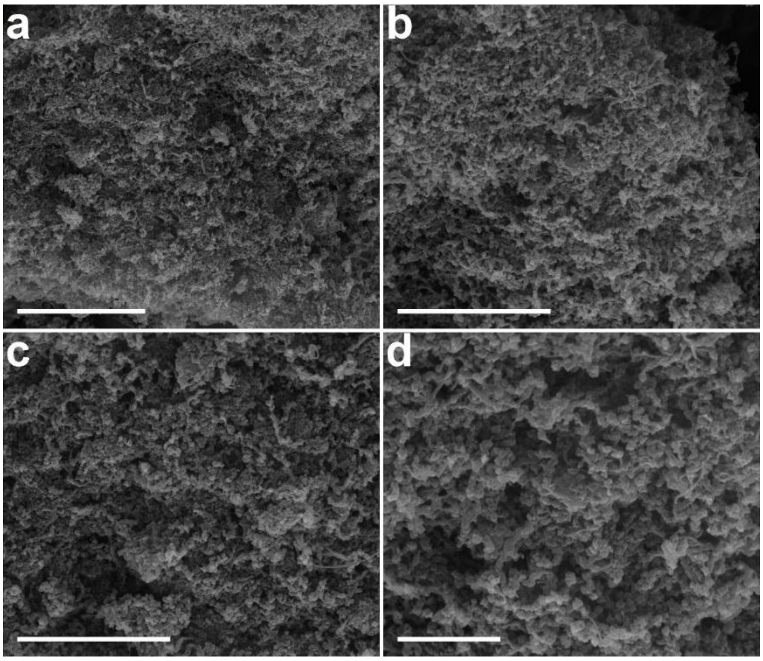

Supplementary Figure 3 | a-d, SEM images of ZnNC at different magnification. Scale bar: **a**, 5 μm; **b**, **c**, 3 μm; **d**, 1 μm.

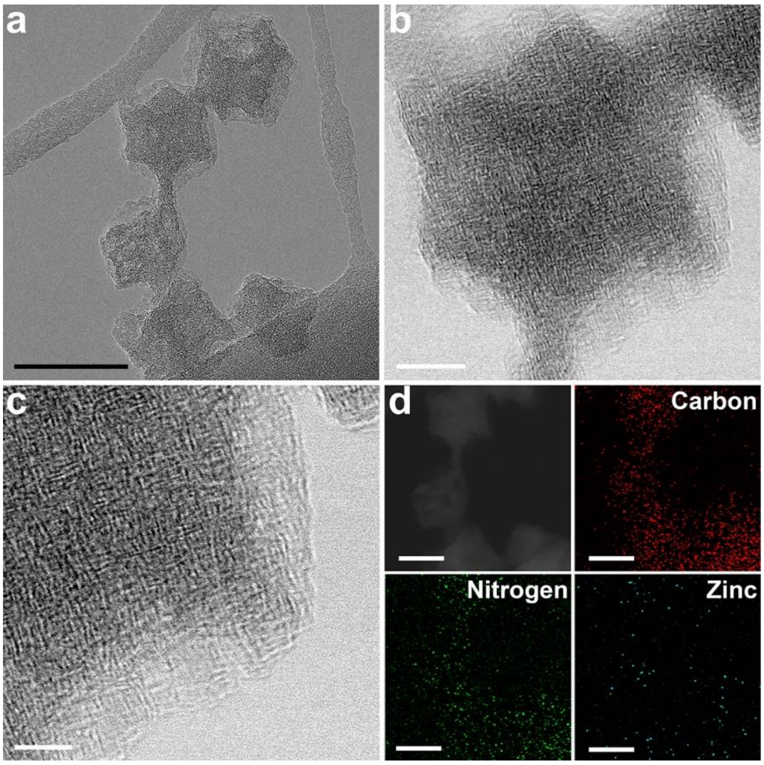

Supplementary Figure 4 | a-c, TEM and STEM images of ZnNC at different magnification. **d**, Corresponding TEM-EDS element mapping images. Scale bar: **a**, 50 nm; **b**, 10 nm; **c**, 5 nm; **d**, 25nm.

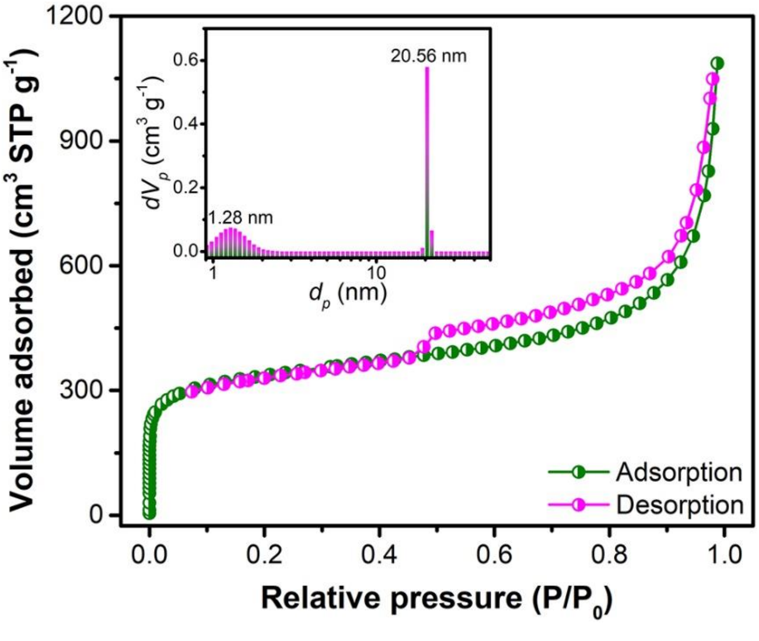

Supplementary Figure 5 | N_2 adsorption/desorption isotherms of ZnNC. Inset is the corresponding pore distribution. The specific surface area is around 1256.7 $m^2\ g^{-1}$.

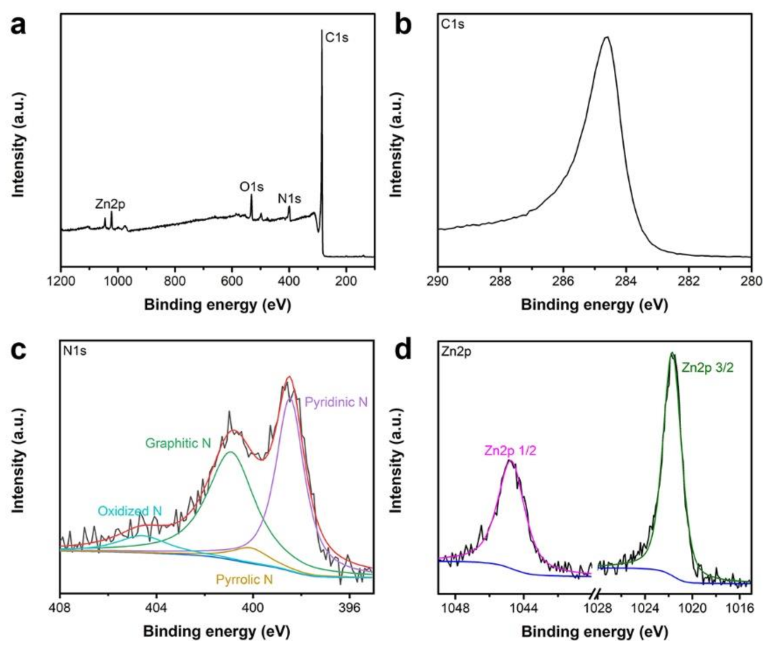

Supplementary Figure 6 | a, Full survey XPS spectrum ZnNC. **b**, High-resolution C 1s spectrum of ZnNC. **c**, High-resolution N 1s spectrum of ZnNC. **d**, High-resolution Zn 2p spectrum of ZnNC.

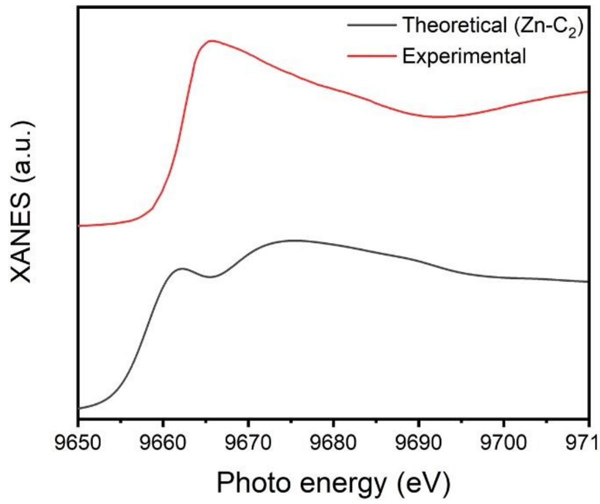

Supplementary Figure 7 | Comparison of Zn K-edge XANES spectrum of ZnNC and theoretical XANES spectrum calculated with Zn-C_2 structure. The Zn-C_2 structure was configured by replacing the nitrogen in Zn-N_2 structure (**Figure 3a**) with carbon.

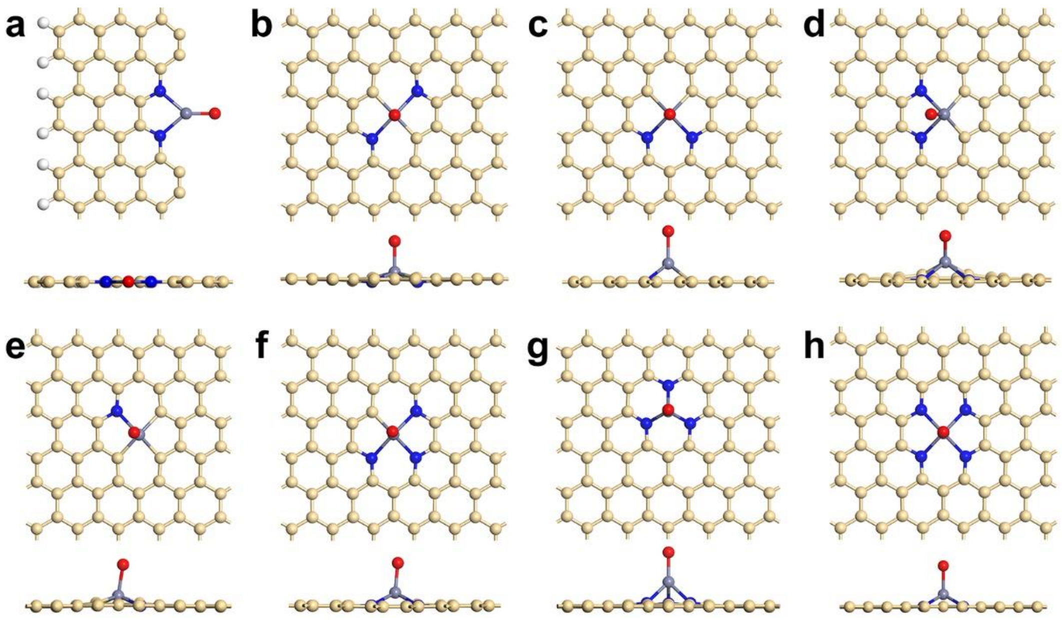

Supplementary Figure 8 | a-h, Top and side views of optimized structures of Zn-N_2, Zn-N_2C_2, Zn-N_1C_3, Zn-N_3C_1, Zn-N_3 and Zn-N_4, respectively.

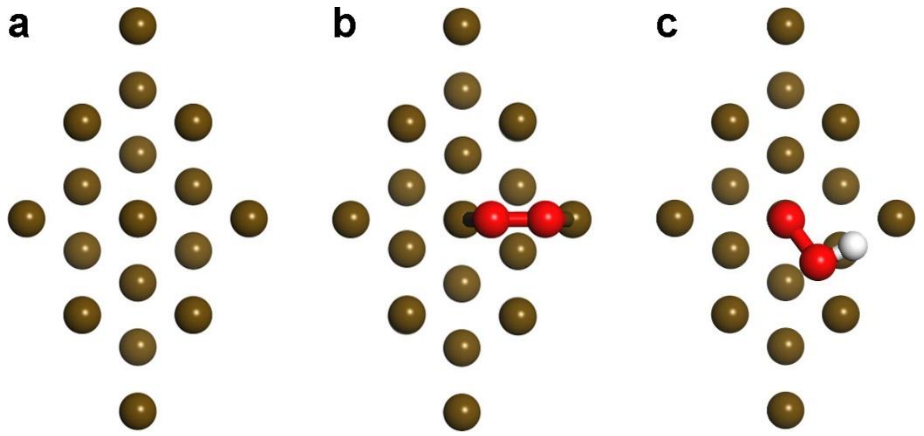

Supplementary Figure 9 | a-c, Optimized structures of Pt(111), O_2 and OOH adsorbed Pt(111). Dark brown, red and white ivory balls represent platinum, oxygen and hydrogen atoms, respectively.

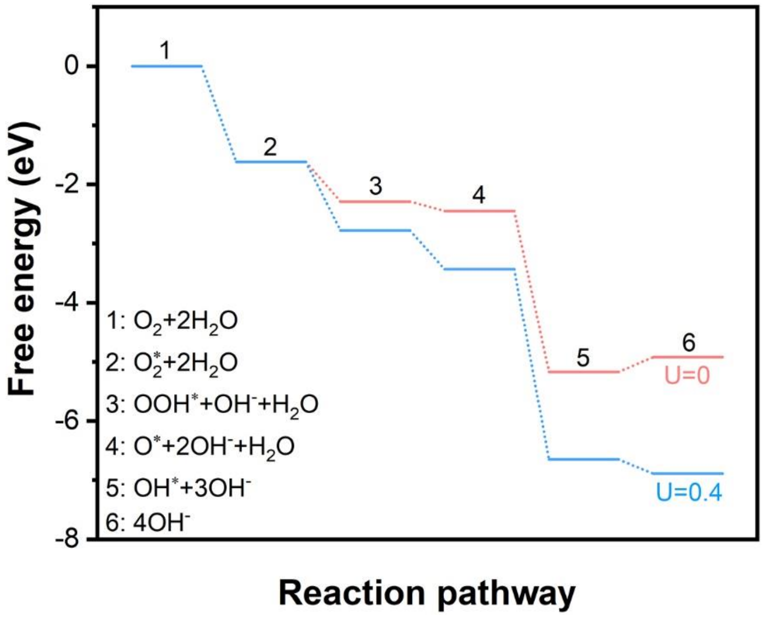

Supplementary Figure 10 | Calculated free energy of oxygen reduction on Zn-N_2 active site at the overpotential of 0 and 0.4 V, respectively.

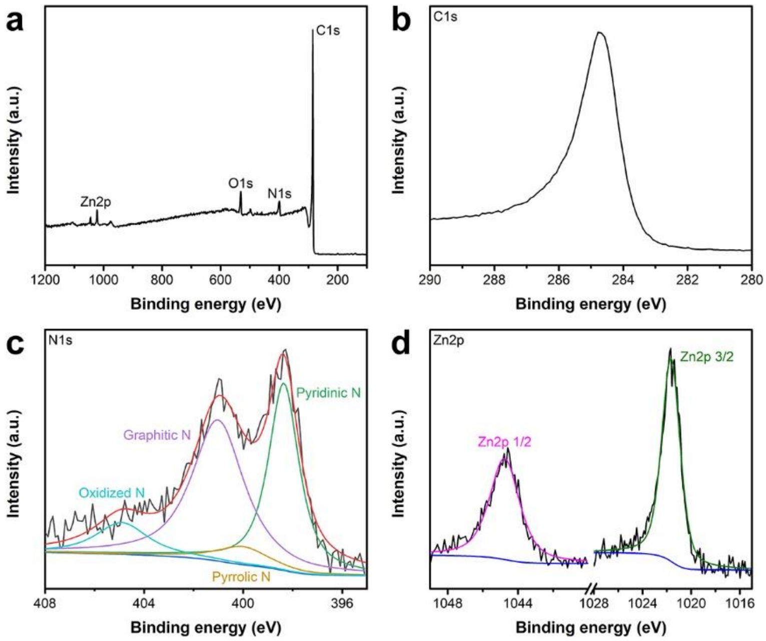

Supplementary Figure 11 | a, Full survey XPS spectrum ZnNC-M. **b**, High-resolution C 1s spectrum of ZnNC-M. **c**, High-resolution N 1s spectrum of ZnNC-M. **d**, High-resolution Zn 2p spectrum of ZnNC-M.

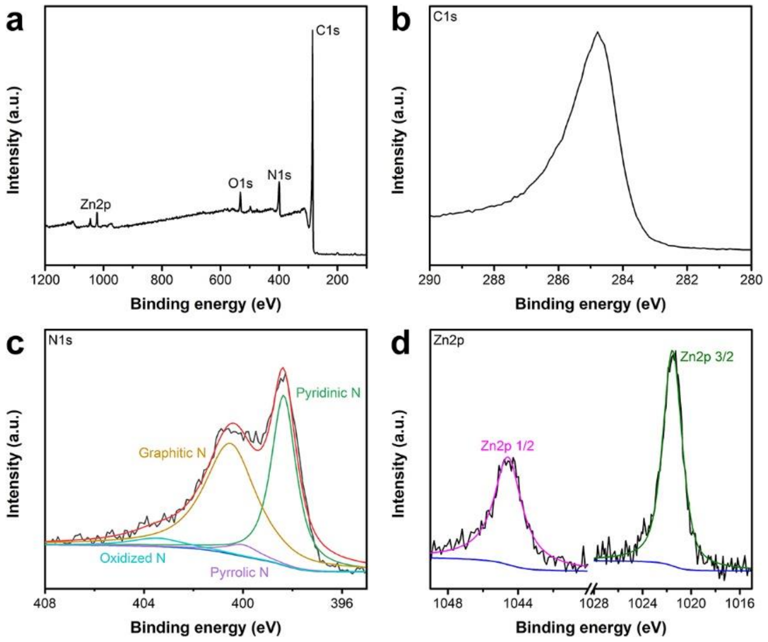

Supplementary Figure 12 | a, Full survey XPS spectrum ZnNC-L. **b**, High-resolution C 1s spectrum of ZnNC-L. **c**, High-resolution N 1s spectrum of ZnNC-L. **d**, High-resolution Zn 2p spectrum of ZnNC-L.

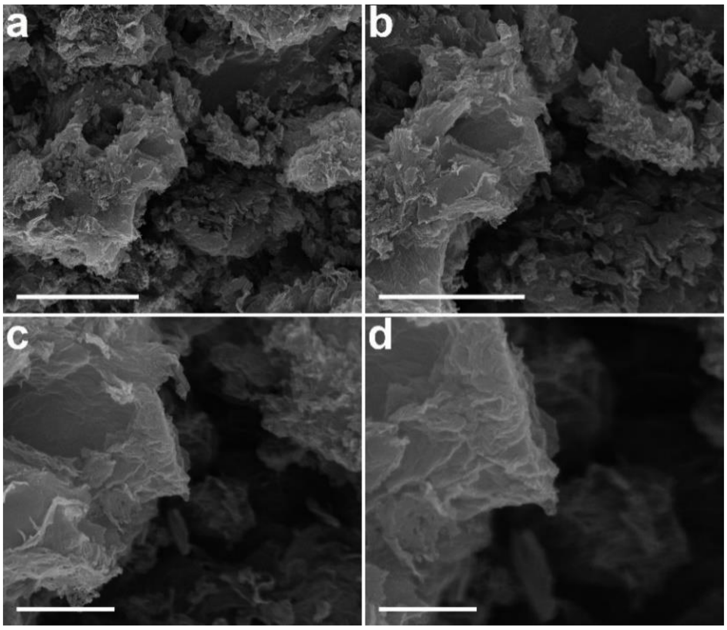

Supplementary Figure 13 | a-d, SEM images of NC at different magnification. Scale bar: **a**, 5 μm; **b**, 3 μm; **c**,1 μm; **d**, 0.5 μm.

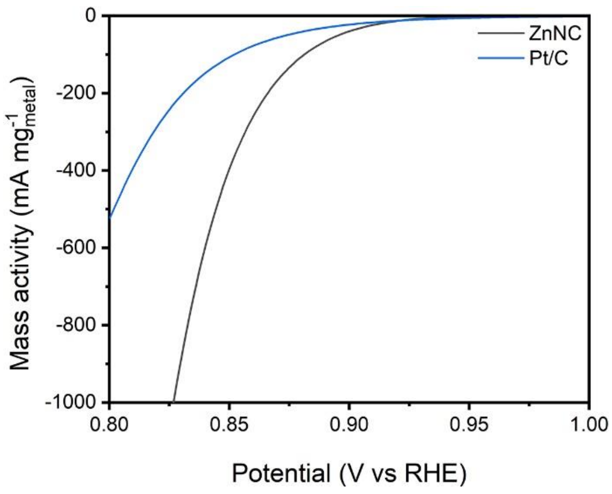

Supplementary Figure 14 | Metal mass normalized polarization curves of ZnNC and Pt/C. The mass activity value of Pt/C and ZnNC at 0.85 V are 106.1 mA $mgPt^{-1}$ and 392 mA $mgZn^{-1}$, respectively. Rotation speed: 1600 r.p.m.; scan rate: 5 mV s^{-1}.

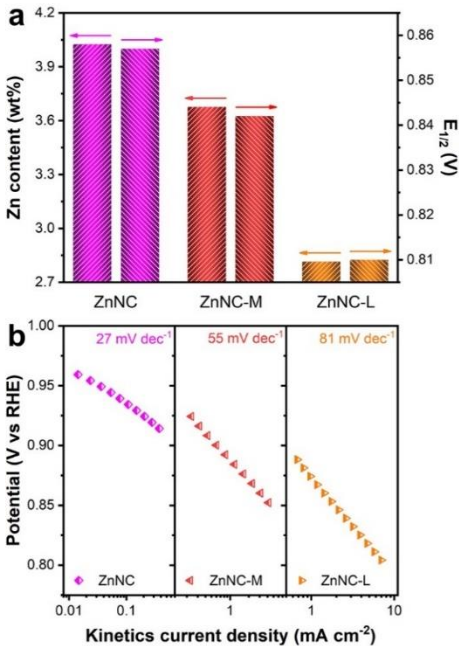

Supplementary Figure 15 | a, $E_{1/2}$s for ZnNC, ZnNC-M and ZnNC-L with difference Zn concentrations. **c**, Tafel plots of potential dependent kinetics current curves of ZnNC, ZnNC-M and ZnNC-L.

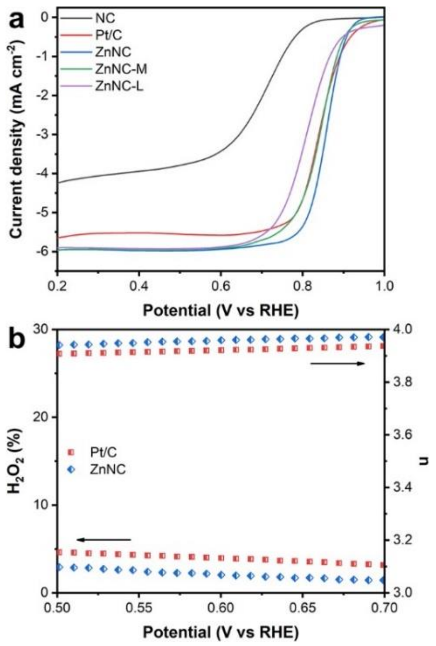

Supplementary Figure 16 | a, Polarization curves of NC, Pt/C, ZnNC, ZnNC-M and ZnNC-L in 0.1 M aq. KOH solution (oxygen saturated). **b**, H_2O_2 yields and n of Pt/C and ZnNC in the potential range of 0.5-0.7 V, respectively. Rotation speed: 1600 r.p.m.; scan rate: 5 mV s^{-1}.

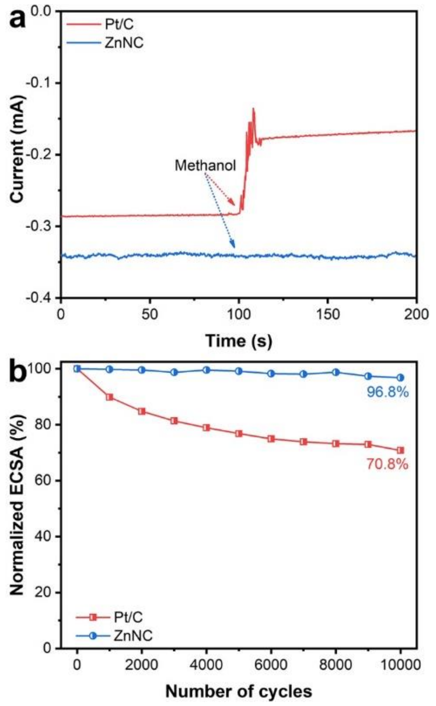

Supplementary Figure 17 | a, Chronoamperometric responses of ZnNC and Pt/C at 0.815 V. The methanol (2 ml) was added in oxygen saturated 0.1 M aq. KOH solution (80 ml) at around 100 s. **b**, Durability test of ZnNC and Pt/C in oxygen saturated 0.1 M aq. KOH solution.

**Supplementary Table 1 | ** Structural parameters extracted from the EXAFS fitting (S^0=1)

Sample	Path	N[a]	R (Å)[b]	σ^2 (10^{-3}Å^2)[c]	ΔE_0 (eV)[d]	R factor
ZnNC	Zn-N	2	1.97	2.8	4.8	0.002
	Zn-O	1	2.05	3.6		

S^0 is the amplitude reduction factor; N is the coordination number; R is bonding distance; σ^2 is Debye-Waller factor; ΔE_0 is energy shift. Fitting range: 2.1 ≤ k (/Å) ≤ 11.6 and 1.0 ≤ R (Å) ≤ 2.0. The structural parameters were obtained by an IFEFFIT program based on FEFF8.

**Supplementary Table 2 | ** Adsorption energies of O_2/OH and O-O bond length of O_2/OOH on Zn-N_2 and Pt(111)

Structure	O_2 (eV)	OH (eV)	O-O (Å, O_2)	O-O (Å, OOH)
**Zn-N_2**	−1.53	−2.71	1.53	1.49
**Pt(111)**	−0.93	−1.93	1.36	1.43

Adsorption energies are calculated by $E_{ads} = E_{product} – E_{reactant}$.

Supplementary Table 3 | ORR parameters for recently reported non-precious metal-based catalysts in alkaline electrolyte

Catalyst	Electrolyte	$E_{1/2}$ (V vs RHE)	Tafel slope (mV dec^{-1})	Reference
**ZnNC**	0.5 mg cm^{-2} (20 μg_{Zn} cm^{-2})	0.857	27	This work
**Co_3O_4/N-rmGO**	0.1 mg cm^{-2} (~51.4 μg_{Co} cm^{-2})	0.83	42	1
**Co_3O_4/rmGO**	0.1 mg cm^{-2} (~51.4 μg_{Co} cm^{-2})	0.79	50	1
**FePhen@MOF-$ArNH_3$**	0.6 mg cm^{-2} (--)	0.86	--	2
**N-CG–CoO**	0.71 mg cm^{-2} (~360 μg_{Co} cm^{-2})	0.81	48	3
**$Co_{0.50}Mo_{0.50}O_yN_z/C$**	0.7 mg cm^{-2} (--)	0.76	71	4
**Fe−N/C−800**	0.1 mg cm^{-2} (~ 2 μg_{Fe} cm^{-2})	0.81	--	5
**rGO-$Cu_{2-x}S$**	-- (~ 64 μg_{Cu} cm^{-2})	0.82	67	6
**Fe-N-CNFs**	0.6 mg cm^{-2} (~ 28 μg_{Fe} cm^{-2})	0.82	--	7
**S,N-Fe/N/C-CNT**	0.6 mg cm^{-2} (~ 20 μg_{Fe} cm^{-2})	0.85	--	8
**Fe_3C@N-CNT**	0.25 mg cm^{-2} (--)	0.85	78	9

Supplementary References

1. Liang, Y. Y. et al. Co_3O_4 nanocrystals on graphene as a synergistic catalyst for oxygen reduction reaction. *Nat. Mater.* **10,** 780-786 (2011).

2. Strickland, K. et al. Highly active oxygen reduction non-platinum group metal electrocatalyst without direct metal-nitrogen coordination. *Nat. Commun.* **6,** 7343 (2015).

3. Mao, S., Wen, Z. H., Huang, T. Z., Hou, Y. & Chen, J. H. High-performance bi-functional electrocatalysts of 3D crumpled graphene-cobalt oxide nanohybrids for oxygen reduction and

evolution reactions. *Energ. Environ. Sci.* **7,** 609-616 (2014).

4. Cao, B. F. et al. Cobalt molybdenum oxynitrides: synthesis, structural characterization, and catalytic activity for the oxygen reduction reaction. *Angew. Chem. Int. Edit.* **52,** 10753-10757 (2013).

5. Lin, L., Zhu, Q. & Xu, A. W. Noble-metal-free Fe-N/C catalyst for highly efficient oxygen reduction reaction under both alkaline and acidic conditions. *J. Am. Chem. Soc.* **136,** 11027-11033 (2014).

6. Wang, X. L. et al. Cu-deficient plasmonic $Cu_{2-x}S$ nanoplate electrocatalysts for oxygen reduction. *ACS Catal.* **5**, 2534-2540 (2015).

7. Wu, Z. Y. et al. Iron carbide nanoparticles encapsulated in mesoporous Fe-N-doped carbon nanofibers for efficient electrocatalysis. *Angew. Chem. Int. Edit.* **54,** 8179-8183 (2015).

8. Chen, P. Z. et al. Atomically dispersed iron-nitrogen species as electrocatalysts for bifunctional oxygen evolution and reduction reactions. *Angew. Chem. Int. Edit.* **56,** 610-614 (2017).

9. Guan, B. Y., Yu, L. & Lou, X. W. A dual-metal-organic-framework derived electrocatalyst for oxygen reduction. *Energ. Environ. Sci.* **9,** 3092-3096 (2016).
